# Assessing effects of modification of middle meatal silastic splint after endoscopic sinus surgery for nasal polyps: A randomized controlled study

**DOI:** 10.1016/j.amsu.2020.08.047

**Published:** 2020-09-06

**Authors:** Haitham Odat, Mohannad Al-Qudah, Firas Alzoubi, Majid Bani-Ata, Shadi Hamouri, Mohammad Al-Alawneh, Mohammad AL-Ameri, Derar Al-Domaidat, Mahmoud Tanash

**Affiliations:** a Division of Otolaryngology, Department of Special Surgery, Faculty of Medicine, Jordan University of Science and Technology, PO Box 3030, Irbid, Jordan; b Department of Surgery, Faculty of Medicine, Jordan University of Science and Technology, PO Box 3030, Irbid, Jordan

**Keywords:** Nasal polyps, Endoscopic sinus surgery, Middle turbinate, Adhesion

## Abstract

**Purpose:**

To investigate the efficacy of middle meatal silastic splint in preventing adhesions after bilateral endoscopic sinus surgery (ESS) for chronic rhinosinusitis with nasal polyps (CRSwNP), and to assess nasal symptoms and endoscopic findings in splinted and non-splinted sides.

**Methods:**

After completion of ESS, silicon silastic splints were randomly inserted in the middle meatus of one nasal side, while no stent in the other side (control). The surgeon was blinded to the side selection, and splint insertion until removal after 1 week. Patients were followed -up after 1 week, 1 and 6 months. Each side of the nasal cavity was assessed for adhesions, crusting, pus, pain, nasal obstruction, and nasal discharge by endoscopic examination and visual analogue scale.

**Results:**

Forty-nine patients (98 nasal sides) were included. At the 1st week visit, there was no significant difference between the splinted and non-splinted sides for all investigated parameters.

After 1- month, adhesions were seen in 10% of the splinted sides, while it was in 26% of the non-splinted sides (P = 0.037).

At the 6 -month follow-up visit, the adhesions rate remained 10% in the splinted sides, however the rate increased to 32% in the non-splinted sides (*P* = 0.007). All other examined parameters remained statistically insignificant between both sides throughout the follow -up visits.

**Conclusions:**

Middle meatal silastic splint is significantly reducing middle meatal adhesions with low complication rate in CRSwNP patients undergoing ESS. Our results support its usage when the middle turbinate is unstable or traumatized during surgery.

## Highlights

• Unstable middle turbinate after endoscopic sinus surgery can cause middle meatal adhesions leading to surgical failure.
• A silastic splints were inserted in the middle meatus of one nasal side, while no stent in the other side.
• The technique is simple, and keeps the middle turbinate lateralized until healing occurs.
• The splint is well tolerated by all patients with no adverse effects.
• Middle meatal silastic splint is significantly reducing middle meatal adhesions with low complication rate.

## 1 Introduction

Chronic Rhinosinusitis (CRS) is an inflammation of the nasal and paranasal sinus mucosa lasting more than 12 weeks. The disease is estimated to affect approximately 13% of the population with a significant impact on patients’ quality of life. It is classified into two phenotypes: CRS with nasal polyps (CRSwNP) and CRS without nasal polyps (CRSsNP) [[1], [2], [3]].

Patients who fail to respond to medical treatments (antibiotics and local steroids) are candidates for endoscopic sinus surgery (ESS) which is considered the gold standard surgical treatment [4]. However, surgical failure can be a significant problem despite advances in operative instrumentations and postoperative management, with reported revision rates between 10% and 66% in the literature [5].

Adhesions often form between middle turbinate and lateral nasal wall after ESS [5,6]. Lateralization of a floppy and unstable middle turbinate, and/or trauma of middle turbinate and lateral nasal wall allow direct contact between the two raw surfaces which eventually lead to adhesions. Synechiae can obstruct the outflow of the maxillary, ethmoid and frontal sinuses leading to persistence or recurrence of symptoms and thus necessitating revision surgery. The incidence of middle meatal adhesions has been reported between 10% and 40% of patients after ESS [6,7].

Numerous absorbable and nonabsorbable materials, like; Floseal, Merocel, Gelfoam, fibrin glue, hyaluronic acid, and mitomycin C have been evaluated for their role in preventing middle meatal

adhesions with variable success rates. However, no method has been accepted as a gold standard technique to fill the middle meatal space after ESS [8].

The aim of this prospective double-blind randomized controlled study was to investigate the efficacy of a silastic splint in preventing synechiae formation between middle turbinate and lateral nasal wall after bilateral ESS for CRSwNP patients, and to assess nasal symptoms and endoscopic middle meatal findings in splinted and non-splinted sides. We also sought to evaluate the patient tolerance of the middle meatal silastic splint.

## 2 Materials and methods

We designed a prospective, double-blind, randomized controlled study for patients with CRSwNP undergoing bilateral ESS. Approval form our institutional review board committee was obtained. All patients were consented before surgery, and they had detailed history, clinical examination including nasal endoscopy and sinonasal CT scan.

Inclusion criteria were adult patients (≥18 years of age), CRwNP who failed to respond to medical treatment (local and systemic steroid, saline irrigation, and antibiotics), bilateral sinus disease, unstable middle turbinate and/or debrided polypoidal middle turbinate mucosa during surgery, and no previous history of endoscopic sinus surgery.

Exclusion criteria included; resected middle turbinate, asymmetric disease involvement, allergic fungal sinusitis, known cases of primary immunodeficiency, granulomatous diseases, cystic fibrosis, primary ciliary dysfunction, patients with unilateral disease, sinonasal malignancy, and patients who did not complete 6 months follow-up time.

Computer-generated randomization (http://www.random.org) was done preoperatively to choose in which side of the nose the silastic splint would be placed, which is referred to as the “splint side”, while the other side (the control side) is referred to as the “non splint side.”

All splints were placed by senior residents. After completion of ESS, the circulating nurse opened a sealed envelope containing the randomization of the stent side to inform the surgeon in which side the splint was to be placed.

**Fig. 1:**
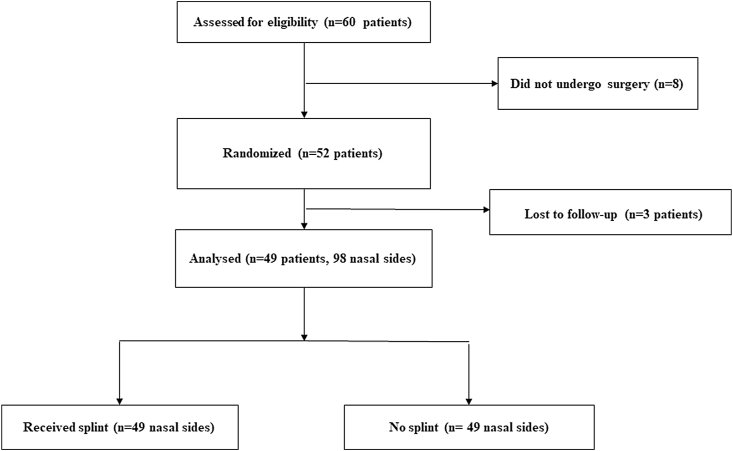
Consolidated Standards of Reporting Trials (CONSORT) flow diagram of the randomized controlled trial.

The protocol of the trial is summarized in a flow diagram according to the Consolidated Standards of Reporting Trials (CONSORT) guidelines (Fig. 1).

**Fig. 2:**
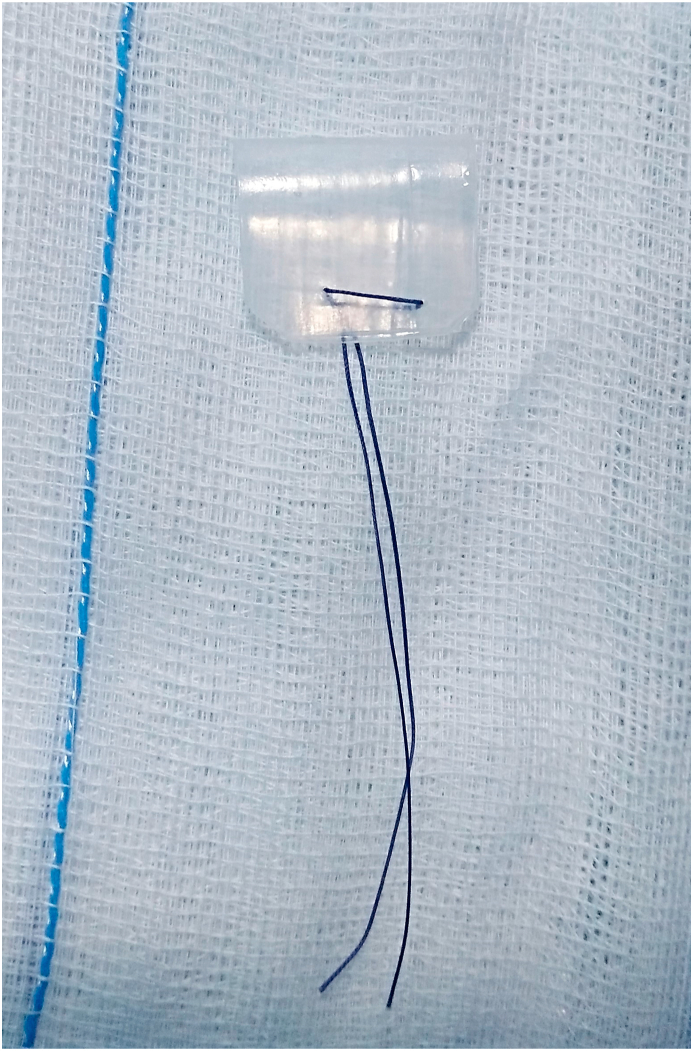
Design of the inverted U-shaped silastic splint with long suture ends.

**Fig. 3:**
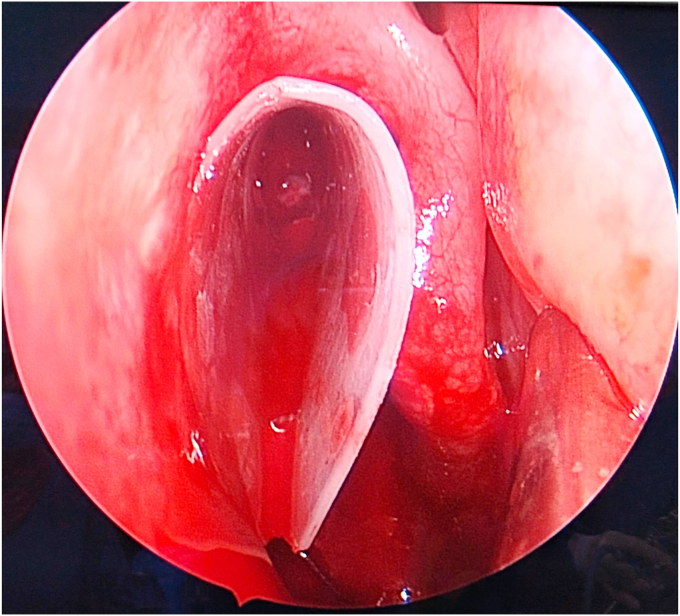
Endoscopic view of a silastic splint in the middle meatus after Endoscopic Sinus Surgery.

All patients were operated by the same surgeon (H·O.). A silicone sheet of 1 mm thickness was used (5 × 5 cm, 0.04 inches; Medtronic, Minneapolis, MN). After cutting the splint to appropriate size, it was folded back on itself and sutured by Vicryl 3/0 (Ethicon, Inc., Cornelia, GA) to form inverted U- shape similar to Gall and Witterick technique [6] but without superior opening and the ends of the suture were cut long for easy removal in the outpatient clinic postoperatively (Fig. 2). The splint size was adjusted to fit in the middle meatus without fixing suture, keeping the anterior border of the splint just outside the anterior border of the middle turbinate and its inferior border below the middle turbinate at the level of the inferior turbinate (Fig. 3). No other middle turbinate medialization techniques were performed and middle meatal packs were not inserted.

All patients were discharged home on the first post-operative day on oral antibiotic for 2 weeks, 20 mg prednisolone/day for 1 week, and saline nasal irrigation q. i.d.

The splint was removed by senior resident at the first postoperative visit after 1 week under local anesthesia for all patients by grasping the long ends of the suture or the anterior border of the splint. Endoscopic examination was performed in the clinic by the same senior surgeon on day 7, 1 month, and 6 months after surgery observing the presence of any synechia, crusting, and pus. Furthermore, the middle meatus of each side was described as “widely open” when the ethmoid cavity easily seen by endoscope compared to the other side.

Each side of the nasal cavity was assessed for pain/discomfort, nasal obstruction, and nasal discharge by using 10 points visual analogue scale (VAS) ranging from 0 (no symptoms) to 10 (maximal symptoms); this was done by ENT resident who was blind to the splinted side.

Sample size was calculated based on the observed middle meatal adhesion rate after ESS in our department of approximately 25%. Recently, Baguley et al. [9] reported an adhesion rate of 3% in sides received middle meatal silastic splint, and 36% in non-splinted sides. Accordingly, a sample size of 38 patients (76 nasal sides) was calculated to achieve 80% power for possible adhesion rates of 3% on the splinted side versus 25% on the non-splinted side, allowing for the probability of a type 1 error of 0.05 between two groups.

Parametric data was analysed using the unpaired *t*-test, while the Mann-Whitney *U* test was used for nonparametric data analysis. For categorical data analysis; the Chi square test or Fisher's exact test was used as appropriate. A P value of <0.05 was considered statistically significant.

## 3 Results

Overall, 60 patients with CRSwNP were recruited but 11 patients were excluded; 8 patients because they did not undergo surgery, and 3 patients did not complete 6 -month follow-up. Forty-nine patients (98 sides) who underwent bilateral ESS for CRSwNP and fulfilled our inclusion and exclusion criteria were included in this study. The mean age was 35 years (range, 18–61 years, SD ± 10). Thirty-one patients (63%) were male and 18 patients (37%) were female. The silastic splint was inserted in the right middle meatus in 22 patients (45%) and in the left side in 27 patients (55%), Table 1.

**Table 1:** Patients demographic data (n = 49).

Age	35 (SD ± 10)
Gender (Male: Female)	31:18
phenotype	Nasal polyps
Splinted side (Right: Left)	22: 27

Interestingly, the patients did not complain of pain/discomfort difference between the splinted and non-splinted sides while the splint was in place. Also, splint removal was well tolerated by all patients and no patient had vaso-vagal attack or significant bleeding necessitated nasal pack. Splint complications such as movement from its place or aspiration were not reported.

The average preoperative VAS for nasal obstruction in the splinted sides was 7.5 (SD ± 2.3), while in the non-splinted sides it was 6.3 (SD ± 2.7). For nasal discharge, the VAS was 3.9 (SD ± 3.2) in the splinted sides versus 3.1(SD ± 3.1) in the non-splinted sides.

At the1^st^ week visit after surgery, there was no statistically significant difference between the splinted and non-splinted sides for all investigated parameters, Table 2.

**Table 2:** One-week postoperative symptom and endoscopic scores.

	Splinted side	Non-splinted side	P value
Pus	5/49 (10%)	6/49 (10%)	0.7
Crusting	29/49 (59%)	27/49 (55%)	0.7
Adhesions	2/49 (4%)	4/49 (8%)	0.4
Pain	1.9 (SD ± 1.6)	1.9 (SD ± 1.3)	0.3
Obstruction	2.6 (SD ± 2.4)	3 (SD ± 2.6)	0.6
Nasal discharge	3.6 (SD ± 2.3)	3.9 (SD ± 1.8)	0.06

After 1- month of surgery, adhesions were seen in 10% of the splinted sides, while it was in 26% of the non-splinted sides with a significant difference (*P* = 0.037). Pain, crusting, pus in the middle meatus, nasal discharge and obstruction were statistically not significant between both sides, Table 3.

**Table 3:** One-month postoperative symptom and endoscopic scores.

	Splinted side	Non-splinted side	P value
Pus	11/49 (22%)	9/49 (18%)	0.6
Crusting	15/49 (31%)	10/49 (20%)	0.2
Adhesions	5/49 (10%)	13/49 (26%)	0.037
Pain	0.44 (SD ± 0.8)	0.32 (SD ± 0.6)	0.08
Obstruction	2.4 (SD ± 2.7)	2.9 (SD ± 2.9)	0.4
Nasal discharge	1 (SD ± 1.4)	1.9 (SD ± 2.6)	0.06

At the 6- month follow-up visit, the adhesions rate remained 10% in the splinted sides, however the rate increased to 32% in the non-splinted sides (*P* = 0.007). All other examined parameters remained statistically insignificant between both sides, Table 4.

**Table 4:** Six-month postoperative symptom and endoscopic scores.

	Splinted side	Non-splinted side	P value
Pus	2/49 (4%)	4/49 (8%)	0.4
Crusting	3/49 (6%)	3/49 (6%)	1
Adhesions	5/49 (10%)	16/49 (32%)	0.007
Pain	0.29 (SD ± 0.7)	0.31 (SD ± 0.8)	0.8
Obstruction	1.74 (SD ± 2)	1.6 (SD ± 2.1)	0.4
Nasal discharge	1.35 (SD ± 2.2)	1.1 (SD ± 2)	0.3

Nasal endoscopic examination showed a widely opened middle meatus in the splinted sides for all patients in comparison to the non-splinted sides throughout all follow-up visits.

## 4 Discussion

Adhesions between the middle turbinate and lateral nasal wall remain the most common complication after ESS with significant impact on surgical success rate [10]. Thick adhesions prevent good sinus ventilation, mucus drainage, and impede topical nasal steroid to reach the middle meatus leading to persistence of symptoms, disease recurrence, and thus revision surgery [11].

In many cases of CRSwNP, the middle turbinate is thin, floppy, unstable, and has polypoidal mucosa that request debridement leaving raw surface. Furthermore, the basal lamella may be eroded by the polyps or during ESS. These factors increase the risk of middle turbinate lateralization and synechiae formation in patients with CRSwNP.

We conducted this prospective double -blind randomized controlled trial to study the effect of silastic splint on reducing middle meatal adhesions after bilateral ESS for nasal polyposis.

In this study we found that middle meatal silastic splint significantly decreased adhesions between middle turbinate and lateral nasal wall. Adhesions was seen in 5 splinted sides (10%) compared to 13 non-splinted sides (26%) after 1-month of surgery (P = 0.037). This incidence remained steady for the splinted sides, while it increased to 32% for the non-splinted sides after 6- months follow-up time.

Although different techniques and packing materials to prevent middle meatal adhesions after ESS have been employed, no universal agreement on their significant efficacy [8].

Making mucosal abrasions between the middle turbinate and nasal septum for permanent medialization of the middle turbinate has been proposed by Friedman and his colleagues [12]. This may compromise airflow to the olfactory region and become difficult to correct if hyposmia/anosmia occurred. Suturing the middle turbinate to the nasal septum is another medialization procedure that has been recommended with variable success rates [13].

Silastic sheet has been used in the middle ear for prevention of adhesions between tympanic membrane and middle ear mucosa with high success rates. It has been found that silastic sheet promotes middle ear mucosa healing without apparent foreign body reaction, rejection, or chronic inflammatory response [14]. The promising role of silastic sheet in reducing middle ear adhesions motivated surgeons to use it in the middle meatus. Gall and Witterick [6] retrospectively reviewed 500 patients who underwent ESS for CRSsNP, CRSwNP, and recurrent sinusitis. Inverted U-shape with superior hole silicon stent was used in 778 sides, while 118 sides were not stented. The stents were removed 2–3 weeks after surgery. They found that adhesions were seen in 4.2% of the stented sides. Twelve patients (2.4%) sneezed out one or both stents before first follow -up visit and in 5 patients (1.1%), the stent was removed earlier than the scheduled time because they complained of severe pain. The retrospective study design and very short follow-up time were the main obvious study limitations.

The ease of the technique with high success rate, and few complications, encouraged other authors to conduct a prospective study using various shapes of silicon stent. Lee and Lee [15] conducted a prospective study on 30 patients (35 sides) who developed an unstable middle turbinate during ESS. Twelve patients had CRSsNP and 18 patients had CRSwNP. They used fan shaped silastic sheet which was sutured to the nasal septum to keep it in place and was removed 2 weeks after surgery. They found that adhesions were seen in 6% of the stented cases compared to 44% in the control group. They concluded that silastic sheet in the middle meatus is an effective method to preserve the middle turbinate when it is floppy or unstable.

A prospective double-blind randomized controlled study was conducted by Baguley et al. [9] to investigate the efficacy of silastic splints in preventing adhesions after bilateral ESS for CRS. Thirty-eight patients were enrolled in the study, 31 patients were operated by one surgeon and 33 patients completed follow-up time of 12 weeks. The silastic splint was placed into one middle meatus and the other side used as control. They shaped the splint from a silastic sheet which was cut with an upper and lower limb that passed on either side of the horizontal portion of the ground lamella to keep it in place. They evaluated the post-operative results by using VAS and video recorded nasal endoscopy. Two weeks after surgery, the splints were removed with low pain score for most of patients. They found adhesions in 32% of the non-splinted sides versus 8% of the splinted sides. After 12 weeks, 27% of the un-splinted sides had adhesions, while no adhesions were found in the splinted sides. They concluded that silastic splint in the middle meatus markedly reduces the incidence of adhesions but increases early obstruction and discomfort. However, symptom and ethmoid cavity scores were not significantly different between splinted and non-splinted sides at 12 weeks follow-up period. Another randomized controlled trial of a middle meatal silastic stent following ESS conducted by Chan et al. [16] on 36 patients who had CRSwNP and CRSsNP. They used inverted U- shape with superior hole silastic stent (like Gall and Witterick [6]). The stent was removed at the 2-week visit and the patients were followed -up for 6 months. They found that the stented side demonstrated a statistically significant decrease in adhesion formation at both 2 weeks and 8 weeks postoperatively. At 6 -month follow-up visits, the nasal endoscopic scores remained equal to those at 8 -week assessment. Interestingly, crusting at the second postoperative week was less in the stented side (27% vs 58%; p < 0.01), but this benefit resolved by the 8-week follow-up (p = 0.62).

All authors who conducted studies on middle meatal silastic splints with various shapes found significant reduction in adhesions rate between splinted and non-splinted sides. Similarly, silastic splints were very well tolerated inside middle meatus and the reported complications rate was very low. Manji and his colleagues [17] conducted a prospective study comparing middle meatal silastic splint with gloved - Merocel in 48 patients. They found that gloved - Merocel spacer had no advantage over the silastic spacer in terms of postoperative healing and middle meatal adhesions although patients reported less pain during gloved - Merocel removal.

We agree with lee and lee [15] that the size of the silastic splint is the most important factor to secure the silastic splint in place and to prevent adhesions as large splint can cause irritation and mucosal injury, while small splint may be displaced or extruded and does not prevent adhesions. However, cutting the splint in an appropriate size is not a difficult procedure to learn and can be done easily even by residents.

The difference in success rates could be explained by: (1) disease selection as all authors included both CRSwNP and CRSsNP in their inclusion criteria, (2) status of the middle turbinate because stable and/or non-traumatized middle turbinate affect synechiae formation, (3) shape of the splint may play a role. In this study, we tried to overcome these factors since all of our patients had CRSwNP and had unstable and/or had debrided polypoidal middle turbinates. Fortunately, we a achieved a high success rate of adhesions free middle meatus in 90% of cases which are close and coincide with reported ones. The splint was well tolerated by all patients with low pain score on VAS which was comparable to the non-splinted side. We did not find a statistically significant difference for pus in the middle meatus, crusting, nasal discharge, and nasal obstruction between the splinted and non-splinted sides throughout the follow-up time of 6 months.

We preferred to use inverted U-shape splint because it takes the curvature of the root of the middle turbinate. An opening in the superior part of the splint was not made as we do not think that the dome of the splint reaches up to level of the frontal recess and interfere with mucus drainage. In addition, a small crust can block the superior opening. The long suture ends in our splint allows easy removal in cases with congested nose with narrow space and even when the ends were retracted, they were pulled anteriorly by suction.

Splint was removed 2 weeks after surgery in all previous reports depending on Lee and Lee [15] description that synechia formation between injured mucosa usually occurs in the first 10–14 days after surgery. In this study, the splint was removed 1-week after surgery and the results were within the same reported range.

## 5 Conclusion

Middle meatal silastic splint is significantly reducing adhesions between the middle turbinate and lateral nasal wall with low complications rate in CRSwNP patients undergoing ESS.

It is well tolerated by patients with insignificant pain and it is cost effective compared to other packing materials. Our results support its usage when the middle turbinate is unstable or traumatized during surgery.

## Ethical approval

This work was approved by the institutional review board committee of King Abdullah University Hospital (Jordan university of Science and Technology), IRB number 26/75/2014.

The authors assert that all procedures contributing to this work comply with the ethical standards of the relevant national and institutional guidelines on human experimentation of King Abdulla University Hospital and with the Helsinki Declaration of 1975, as revised in 2008.

## Funding

No fund was received.

## Guarantor

Haitham Odat.

## Informed consent

Informed consents were obtained from all patients.

## Author contribution

All authors contributed significantly in study design, data collection, data analysis, drafting the manuscript, and revising the study.

## Provenance and peer review

Not commissioned, externally peer reviewed.

## Registration of Research Studies

1 Name of the registry: Research Registry.
2 Unique Identifying number or registration ID: researchregistry5855.
3 Hyperlink to your specific registration (must be publicly accessible and will be checked):

https://www.researchregistry.com/browse-the-registry#home/

## Declaration of competing interest

No conflict of interest to declare.

## References

[1] Fokkens W.J., Lund V.J., Hopkins C. (2020). European position paper on rhinosinusitis and nasal polyps 2020. Rhinology.

[2] Benjamin M.R., Stevens W.W., Li N. (2019). Clinical characteristics of patients with chronic rhinosinusitis without nasal polyps in an academic setting. J Allergy Clin Immunol Pract.

[3] Gliklich R.E., Metson R. (1995). The health impact of chronic sinusitis in patients seeking otolaryngologic care. Otolaryngol. Head Neck Surg..

[4] Wright E.D., Agrawal S. (2007). Impact of perioperative systemic steroids on surgical outcomes in patients with chronic rhinosinusitis with polyposis: Evaluation with the novel Perioperative Sinus Endoscopy (POSE) scoring system. Laryngoscope.

[5] Mendelsohn D., Jeremic G., Wright E.D. (2011). Revision rates after endoscopic sinus surgery: A recurrence analysis. Ann. Otol. Rhinol. Laryngol..

[6] Gall R.M., Witterick I.J. (2004). The use of middle meatal stents post-endoscopic sinus surgery. J. Otolaryngol..

[7] Chambers D.W., Davis W.E., Cook P.R., Nishioka G.J., Rudman D.T. (1997). Long-term outcome analysis of functional endoscopic sinus surgery: correlation of symptoms with endoscopic examination findings and potential prognostic variables. Laryngoscope.

[8] Orlandi R.R., Lanza D.C. (2004). Is nasal packing necessary following endoscopic sinus surgery?. Laryngoscope.

[9] Baguley C.J., Stow N.W., Weitzel E.K., Douglas R.G. (2012). Silastic splints reduce middle meatal adhesions after endoscopic sinus surgery. Am J Rhinol Allergy.

[10] Musy P.Y., Kountakis S.E. (2004). Anatomic findings in patients undergoing revision endoscopic sinus surgery. Am. J. Otolaryngol..

[11] Ramadan H.H. (1999). Surgical causes of failure in endoscopic sinus surgery. Laryngoscope.

[12] Friedman M., Landsberg R., Tanyeri H. (2000). Middle turbinate medialization and preservation in endoscopic sinus surgery. Otolaryngol. Head Neck Surg..

[13] Thornton R.S. (1996). Middle turbinate stabilization technique in endoscopic sinus surgery. Arch. Otolaryngol. Head Neck Surg..

[14] Ng M., Linthicum F.H. (1992). Long-term effects of Silastic sheeting in the middle ear. Laryngoscope.

[15] Lee J.Y., Lee S.W. (2007). Preventing lateral synechia formation after endoscopic sinus surgery with a silastic sheet. Arch. Otolaryngol. Head Neck Surg..

[16] Chan C.L., Elmiyeh B., Woods C. (2015). A randomized controlled trial of a middle meatal silastic stent for reducing adhesions and middle turbinate lateralization following endoscopic sinus surgery. Int Forum Allergy Rhinol.

[17] Manji J., Habib A.R., Macias-Valle L. (2018). Comparing the efficacy of Silastic and gloved-Merocel middle meatal spacers for functional endoscopic sinus surgery: A randomized controlled trial. Int Forum Allergy Rhinol.

